# Targeting physiotherapy resources - evaluation of the southampton physiotherapy post-operative screening tool (sppost)

**DOI:** 10.1186/2197-425X-3-S1-A549

**Published:** 2015-10-01

**Authors:** J Weblin, DJ McWilliams

**Affiliations:** UHB NHS Foundation Trust, Birmingham, United Kingdom

## Introduction

The benefit and necessity for prophylactic physiotherapy post operatively remains unclear [1]. Combined with an increased demand on resources, scores to identify those patients who would most benefit are being increasingly used. The SPPOST is a tool to identify patients who are at high risk of developing post-operative pulmonary complications (PPC’s). A previous trial of its implementation demonstrated physiotherapy to be safely withdrawn from 46% of post-operative patients with minimal risk [2].

## Objectives

To assess the feasibility of the SPPOST as an effective tool in prioritising patients for post-operative physiotherapy treatment within a large UK based specialist surgery service.

## Methods

All patients undergoing abdominal surgical procedures between 29^th^ August and 5^th^ October 2013 were included in the analysis. Patients were excluded if they had their operation at a weekend. SPPOST scores were calculated on the first post-operative day, with a score ≥10 identifying a patient as high risk and requiring physiotherapy assessment and intervention. Physiotherapists were blinded to the threshold score and treatment was delivered based on individual clinical reasoning. Patients were assessed daily for the development of a PPC using the Brooks-Brunn criteria. Data was analysed using the Chi squared test.

## Results

Ninety three patients were included in the analysis, with 54 (58%) deemed high risk. More PPC’s were identified in the high risk patients, although this was not significant (9, 17% vs 3, 8%; p = 0.20).A subsequent analysis identified 16 low risk patients having received physiotherapy, whilst 14 high risk patients were not assessed by a physiotherapist. It was noted junior members of staff were more likely to screen out high risk patients.

## Conclusions

A threshold score of 10 appeared to be sensitive for identifying patients at higher risk of developing PPC’s. Although not statistically significant, it is acknowledged as a pilot project that the study may not have been appropriately powered. A sub analysis of the data suggested the SPPOST could provide a structured and robust approach to identifying post-operative patients who would most benefit from physiotherapy, particularly for junior members of staff. It is acknowledged that a number of patients in the low risk category still received physiotherapy, which may have reduced the incidence of PPC’s observed. Appropriately powered trials using the SPPOST as a screening tool for physiotherapy input are therefore needed to confirm its effectiveness.
